# Unbiased screening reveals that blocking exportin 1 overcomes resistance to PI3Kα inhibition in breast cancer

**DOI:** 10.1038/s41392-019-0085-2

**Published:** 2019-11-22

**Authors:** Xue-Ling Liu, Bo-Bo Wang, Yi Wang, Yu-Xiang Wang, Chun-hao Yang, Cun Tan, Xi Zhang, Qiao-jun He, Jian Ding, Ling-Hua Meng

**Affiliations:** 1 Division of Anti-Tumor Pharmacology, Shanghai Institute of Materia Medica, Chinese Academy of Sciences, 501 Haike Road, Shanghai, 201203 China; 2 Zhejiang Province Key Laboratory for Drug Evaluation and Clinical Research, First Affiliated Hospital, School of Medicine, Zhejiang University, 79 Qingchun Road, Hangzhou, 310003 China; 3 Zhejiang Province Key Laboratory of Anti-Cancer Drug Research, College of Pharmaceutical Sciences, Zhejiang University, 866 Yuhangtang Road, Hangzhou, 310058 China; 4 University of Chinese Academy of Sciences, No. 19A Yuquan Road, Beijing, 100049 China; 5 Department of Medicinal Chemistry, Shanghai Institute of Materia Medica, Chinese Academy of Sciences, 555 Zuchongzhi Road, Shanghai, 201203 China; 6 Division of Anti-Tumor Pharmacology, State Key Laboratory of Drug Research, Shanghai Institute of Materia Medica, Chinese Academy of Sciences, 555 Zuchongzhi Road, Shanghai, 201203 China

**Keywords:** Breast cancer, Drug screening

**Dear Editor,**

Targeting PI3K is a promising approach for cancer therapy, and the PI3Kα-selective inhibitor alpelisib has been approved for breast cancer treatment.^1–3^ However, the development of acquired resistance poses a significant clinical challenge. Loss of PTEN and activation of mTOR, CDK4/6, or PIM have been reported to mediate acquired resistance to alpelisib.^4–7^ The mechanisms leading to resistance to PI3Kα inhibitors appear to be different under different circumstances, and the aforementioned strategies may be beneficial for a particular group of patients. New strategies to overcome acquired resistance in a broad spectrum of patients need to be discovered.

CYH33 is a novel PI3Kα-selective inhibitor in phase I clinical trials (NCT03544905) and exhibits potent activity against breast cancer in preclinical settings.^8^ Given that the structure of CYH33 distinct from that of alpelisib, the mechanisms of acquired resistance to CYH33 may be unlike those of alpelisib. To monitor the occurrence of resistance to CYH33, we established CYH33-resistant cell lines named MCF7R (Fig. 1a and Fig. S1a, b) and T47DR (Fig. S2a) by exposing human breast cancer MCF7 and T47D cells to increasing concentrations of CYH33.

**Fig. 1:**
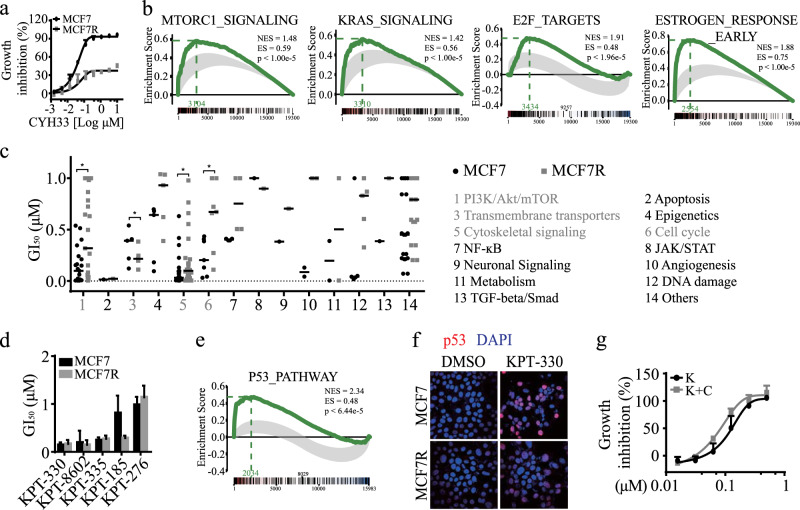
XPO1 inhibition overcame acquired resistance to CYH33. **a** The proliferation of indicated cells treated with CYH33 was evaluated with an SRB assay. **b** Gene sets that were enriched in MCF7R cells compared to those in parental cells, as analyzed with GSEA. **c** GI_50_ of the indicated inhibitors in MCF7R and parental cells; the mean of two independent experiments is shown. The *p* value was calculated by a Student’s *t* test. *p < 0.05. **d** Cells were treated with the indicated XPO1 inhibitors, and the GI_50_ was evaluated. **e** Gene sets that were enriched in MCF7R cells treated with KPT-330 compared to those in cells treated with DMSO, as analyzed with GSEA. **f** Cells were treated with KPT-330 (1 μM) for 24 h and were processed for an immunofluorescence assay with an antibody against p53. **g** MCF7R cells were treated with the indicated inhibitors for 72 h, and cell proliferation was measured. K: KPT-330; C: CYH33. Data shown are the mean ± SD or representative of three independent experiments.

To elucidate the mechanisms of acquired resistance to CYH33, we examined PI3K signaling upon CYH33 treatment in both resistant and parental cells. As shown in Fig. S1c, CYH33 suppressed the phosphorylation level of the PI3K downstream effector Akt in both resistant and parental cells in a dose-dependent manner, suggesting that acquired resistance was not due to the lack of inhibition of its cellular target. However, CYH33 barely altered the activation of mTORC1 substrates, such as p-S6K1 and p-4EBP1, in MCF7R cells, while the phosphorylation of both proteins declined in the presence of CYH33 in their parental counterparts, indicating that mTOR might remain activated in a PI3K-independent manner in cells resistant to CYH33.

To further explore the mechanisms of acquired resistance to CYH33, global gene-expression profiling was performed in MCF7R and parental cells by microarray analysis. Differentially expressed genes (fold change cutoff of 1.5-fold) are presented in Fig. S1d, demonstrating a distinct gene-expression profile of CYH33-resistant cells compared to parental cells. GSEA analysis revealed that the gene signatures of multiple oncogenic pathways, such as mTORC1 signaling, E2F targets, KRAS signaling, and the estrogen response, were upregulated in MCF7R cells compared to their parental counterparts (Fig. 1b), indicating that multiple proliferative signaling pathways might mediate resistance to CYH33.

As multiple signaling pathways involved in cell proliferation were upregulated in MCF7R cells and targeting a single pathway is unlikely to overcome the resistance induced by CYH33, we performed unbiased screening with 2322 agents targeting the hallmarks of cancer, such as cell proliferation, metabolism, and apoptosis, to identify potential therapeutic strategies to overcome resistance to CYH33. There were 83 agents that inhibited the proliferation of MCF7R cells by more than 70% at 1 μM, and the GI_50_s of these compounds against MCF7R cells and their parental counterparts were determined and are shown in Fig. 1c. Agents targeting PI3K/AKT/mTOR, cytoskeletal signaling, and the cell cycle were less potent in CYH33-resistant cells, which was consistent with the observation that multiple oncogenic pathways were activated in resistant cells. Interestingly, compounds targeting transmembrane transporters, especially XPO1, significantly inhibited the proliferation of MCF7R cells, with lower GI_50_s in resistant cells than in parental cells (Fig. 1c). In addition, XPO1 inhibition downregulated most of the gene sets that were altered in MCF7R cells (Fig. S3a), indicating that targeting transmembrane transporters might overcome the resistance to PI3Kα inhibition. XPO1 is responsible for the nuclear export of many tumor suppressor proteins and cell growth regulators and has been validated as an important target for cancer therapy.^9^ XPO1 overexpression was also associated with poor relapse-free survival in breast cancer patients (Fig. S1e). The protein levels of XPO1 remained unchanged in MCF7R and parental cells (Fig. S3c), and a panel of XPO1-selective inhibitors was able to overcome resistance to CYH33 in both MCF7R cells (Fig. 1d) and T47DR cells (Fig. S2b, c).

To investigate the mechanisms of XPO1 inhibition that overcome the resistance to CYH33 in breast cancer cells, we examined the expression profiles of MCF7R and parental cells upon treatment with KPT-330, one of the most advanced XPO1 inhibitors, with a gene-expression microarray. GSEA analysis revealed that genes related to the p53 pathway were upregulated in MCF7R cells upon KPT-330 treatment (Fig. 1e). p53 can be exported by XPO1 from the nucleus to the cytoplasm, and inhibition of XPO1 has been reported to result in nuclear accumulation of p53 and cell cycle arrest.^10^ Indeed, p53 accumulated in the nucleus after MCF7R cells were treated with KPT-330 (Fig. 1f and Fig. S3b).

As CYH33 downregulated the phosphorylation level of Akt in CYH33-resistant cells (Fig. S1c), we investigated the effect of the concurrent inhibition of PI3K and XPO1 on cells with acquired resistance to CYH33. Though CYH33 at 0.3 μM displayed little activity against the proliferation in MCF7R cells (Fig. 1g) or T47DR cells (Fig. S2d), addition of CYH33 to serially diluted KPT-330 significantly enhanced the activity of KPT-330, with a CI of 0.68 in MCF7R cells and 0.35 in p53-mutated T47DR cells, indicating that these inhibitors displayed synergistic activity.

In summary, continuous exposure to the PI3Kα inhibitor CYH33 resulted in the activation of multiple oncogenic pathways that were independent of PI3K. The inhibition of XPO1 overcame the resistance to CYH33 at least partially by accumulating p53 in the nucleus, while the combination of KPT-330 and CYH33 exhibited synergistic antiproliferative activity in CYH33-resistant cells irrespective of p53 mutations. These results provided insight into the mechanisms leading to resistance to CYH33 and provided a rational strategy to enhance the efficacy of CYH33 in ongoing clinical trials.

## Supplementary information

Supplemental material

Revised Supplemental information
