# Supplementary material for: Unbiased screening reveals that blocking exportin 1 overcomes resistance to PI3Kα inhibition in breast cancer
Source: Signal Transduct Target Ther. 2019 Nov 22;4:49. doi: 10.1038/s41392-019-0085-2 (PMC6872586; doi:10.1038/s41392-019-0085-2)
Supplement: Supplementary file 1 — Supplemental material [file 41392_2019_85_MOESM1_ESM.docx]

**Supplemental Information**

**Materials and methods**

**Compounds**

CYH33 was synthesized and provided by Dr. Chun-hao Yang (Shanghai Institute of Materia Medica, Chinese Academy of Sciences, Shanghai, China). All the other compounds used in this study were purchased from Selleck Chemicals (Houston, USA). All compounds were dissolved in dimethyl sulfoxide (Sigma-Aldrich, St. Louis, MO, USA) at 10 mM as stock solutions and stored at -20℃.

**Cell lines**

Human breast cancer MCF7 cells were obtained from American Type Culture Collection (Manassas, VA, USA) and authenticated by analyzing short-tandem repeats (STR) by Genesky Biotechnologies Inc. (Shanghai, China). To generate CYH33-resistant cell lines, MCF7 cells were treated with increasing concentrations of CYH33 (starting at the concentration to induce cell growth inhibition by 20%) until cells proliferate in the presence of 10 μM CYH33. These cell lines were cultured in DMEM supplemented with 10% FBS (Gibco, Grand Island, NY).

**Cell proliferation assay**

The effects of compounds on cell proliferation were evaluated by Sulforhodamine B (SRB; Sigma-Aldrich, St. Louis, MO, USA) assay. Combination Index (CI) was calculated by the ratio of IC_50_ obtained with the combination of KPT-330 and CYH33 to that with KPT-330 alone. If CI < 1, the drugs act synergistically; if CI = or > 1, the drug interaction is defined as additive or antagonistic respectively.

For colony formation assay, cells were seeded in 12-well plates at a density of 1000 cells per well and treated with compounds for 10 days. Colonies were stained with SRB and colonies with a diameter ≥ 1 mm were counted.

**Western blot**

Cell lysates were collected and subjected to standard Western blot analysis as described previously32 with antibodies against phospho-Akt (Ser473), Akt, phospho-S6 Kinase (S6K, Thr389), S6K, phospho-S6 Ribosomal Protein (S6, Ser235/236), phospho-S6 (Ser240/244), S6, phospho-4EBP1 (Thr37/46), 4EBP1 (Cell Signaling Technology, Danvers, MA, USA), β-actin (Sigma-Aldrich, St. Louis, MO, USA).

**Microarray analysis**

The microarray analysis was conducted at Shanghai Baygene Biotechnology Co. Ltd (Shanghai, China). Total RNA was extracted and subjected to gene expression analysis with Affymetrix Human PrimeView microarrays (Santa Clara, CA, USA) according to the manufacturer’s instructions. Molecular pathway analysis was performed using Gene Set Enrichment Analysis (GSEA, <http://software.broadinstitute.org/gsea/index.jsp>).

**Immunofluorescence assay**

Cells grown on coverslips were washed with phosphate-buffered saline (PBS) and fixed with 4% paraformaldehyde (Melonepharma, Dalian, China) for 15 min. Cells were then permeabilized with 0.2% Triton X-100 (Sigma-Aldrich) in PBS for 8 min, blocked with PBS containing 3% BSA for 30 min and incubated with antibodies against p53 overnight. After being washed with PBS, cells were incubated with Alexa Fluor 633-conjugated secondary antibodies (Invitrogen, Carlsbad, CA, USA) for 2 h, and then stained with DAPI (Vector Laboratories, Burlingame, CA, USA). Images of cells were acquired with Olympus BX51 fluorescence microscope (Olympus, Japan).

**Statistical analysis**

Data presented were from at least two or three independent experiments. Statistical analysis was performed as indicated. Differences were considered statistically significant when p value was less than 0.05.

**Supplemental Figure 1**


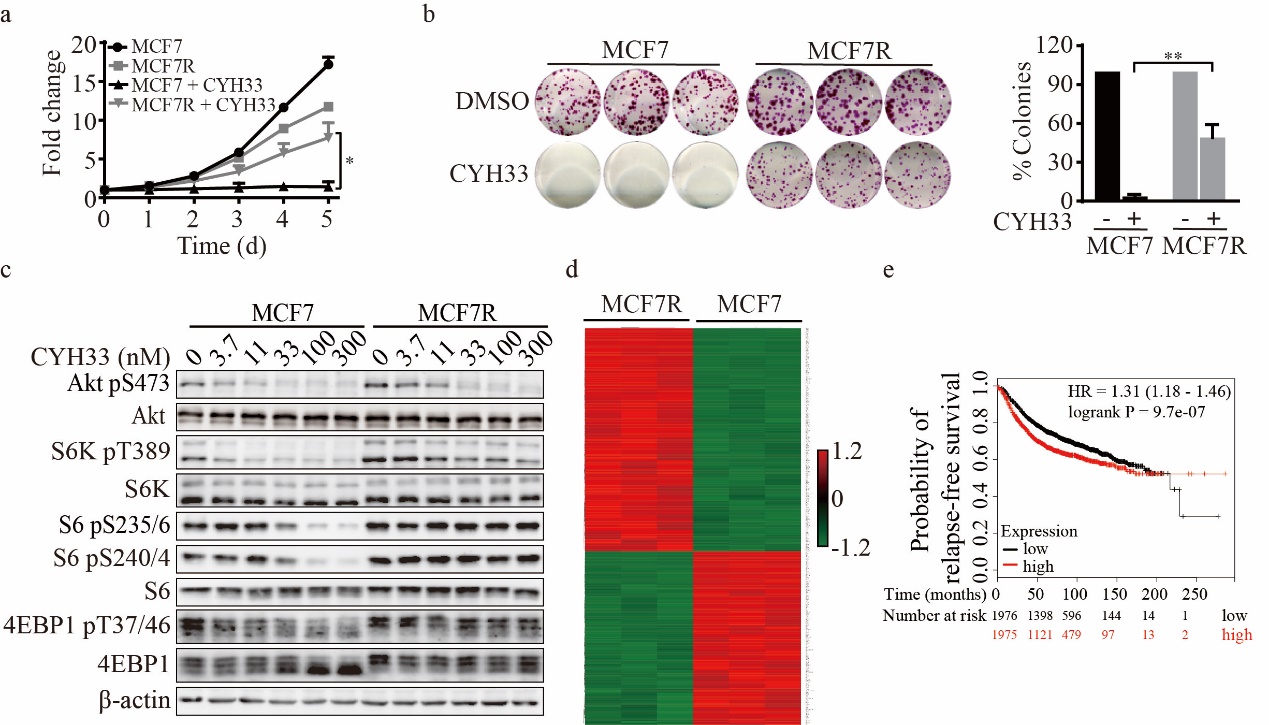


(a) Cells were treated with CYH33 (1 μM) or DMSO and cell proliferation was assessed by SRB assay at indicated times. (b) Representative images of colony formation of indicated cells treated with CYH33 (0.3 μM) or DMSO and the colonies formed were quantitated. (c) Cells were treated with CYH33 for 24 h and cell lysates were subjected to Western blot with indicated antibodies. (d) The heatmap of genes differentially expressed with statistical significance in MCF7R and parental cells. Biological replicates = 3. (e) The relationship between the expression level of XPO1 and the probability of relapse-free survival (RFS) in breast cancer patients was analyzed with Kaplan-Meier Plotter. Data shown in (a & b) were mean + SD. p value in (a & b) was calculated by Student’s t-test. **: p < 0.01; *: p < 0.05.
