# Supplementary material for: Unbiased screening reveals that blocking exportin 1 overcomes resistance to PI3Kα inhibition in breast cancer
Source: Signal Transduct Target Ther. 2019 Nov 22;4:49. doi: 10.1038/s41392-019-0085-2 (PMC6872586; doi:10.1038/s41392-019-0085-2)
Supplement: Supplementary file 2 — Revised Supplemental information [file 41392_2019_85_MOESM2_ESM.docx]

Supplementary Materials for

An unbiased screening reveals blocking exportin 1 overcomes resistance to PI3Kα inhibition in breast cancer

Xue-ling Liu^1,2^, Bo-bo Wang^1,3^, Yi Wang^1^, Yu-xiang Wang^1,4^, Chun-hao Yang^5^, Cun Tan^5^, Xi Zhang^1^, Qiao-jun He^3^, Jian Ding^4,6^ & Ling-hua Meng^1,4^

^1^ Division of Anti-tumor Pharmacology, Shanghai Institute of Materia Medica, Chinese Academy of Sciences, 501 Haike Road, Shanghai 201203, China; ^2^ Zhejiang Province Key Laboratory for Drug Evaluation and Clinical Research, First Affiliated Hospital, School of Medicine, Zhejiang University, 79 Qingchun Road, Hangzhou 310003, China;^3^ Zhejiang Province Key Laboratory of Anti-Cancer Drug Research, College of Pharmaceutical Sciences, Zhejiang University, 866 Yuhangtang Road, Hangzhou 310058, China;^4^ University of Chinese Academy of Sciences, No.19A Yuquan Road, Beijing 100049, China;^5^ Division of Anti-tumor Pharmacology, State Key Laboratory of Drug Research, Shanghai Institute of Materia Medica, Chinese Academy of Sciences, 555 Zuchongzhi Road, Shanghai 201203, China; ^6^ Department of Medicinal Chemistry, Shanghai Institute of Materia Medica, Chinese Academy of Sciences, 555 Zuchongzhi Road, Shanghai 201203, China.

These authors contributed equally: Xue-ling Liu, Bo-bo Wang.

Correspondence to: Jian Ding ([jding@simm.ac.cn](mailto:jding@simm.ac.cn)) or Ling-hua Meng (lhmeng@simm.ac.cn).

**This PDF file includes:**

Materials and Methods

Supplementary Text

Figures. S1 to S3

Materials and Methods

Compound

CYH33 was synthesized and provided by Dr. Chun-hao Yang (Shanghai Institute of Materia Medica, Chinese Academy of Sciences, Shanghai, China). All the other compounds used in this study were purchased from Selleck Chemicals (Houston, USA). All compounds were dissolved in dimethyl sulfoxide (Sigma-Aldrich, St. Louis, MO, USA) at 10 mM as stock solutions and stored at -20℃.

Cell lines

Human breast cancer MCF7 and T47D cells were obtained from American Type Culture Collection (Manassas, VA, USA) and authenticated by analyzing short-tandem repeats (STR) by Genesky Biotechnologies Inc. (Shanghai, China). MCF7 cells were cultured in DMEM supplemented with 10% FBS (Gibco, Grand Island, NY). T47D cells were cultured in RPMI 1640 supplemented with 10% FBS (Gibco). To generate CYH33-resistant cell lines, MCF7 and T47D cells were treated with increasing concentrations of CYH33 (starting at the concentration to induce cell growth inhibition by 20%) until cells proliferated in the presence of 10 μM CYH33.

Cell proliferation assay

The effects of compounds on cell proliferation were evaluated by Sulforhodamine B (SRB; Sigma-Aldrich, St. Louis, MO, USA) assay. In single agent assay, the inhibitory rate was calculated as (OD540 control - OD540 compound)/OD540 control * 100%, while the inhibitory rate of drug combination was calculated as (OD540 _CYH33_ - OD540 combination)/OD540 _CYH33_ * 100%. Combination Index (CI) was calculated by the ratio of IC_50_ obtained with the combination of KPT-330 and CYH33 to that with KPT-330 alone with the formula: CI = IC_50_ (KPT-330 plus CYH33) / IC_50_ (KPT-330). If CI < 1, the drugs act synergistically; if CI = or > 1, the drug interaction is defined as additive or antagonistic respectively.

For colony formation assay, cells were seeded in 12-well plates at a density of 1000 cells per well and treated with compounds for 10 days. Colonies were stained with SRB and colonies with a diameter ≥ 1 mm were counted.

Western blot

Cell lysates were collected and subjected to standard Western blot analysis with antibodies against phospho-Akt (Ser473), Akt, phospho-S6 Kinase (S6K, Thr389), S6K, phospho-S6 Ribosomal Protein (S6, Ser235/236), phospho-S6 (Ser240/244), S6, phospho-4EBP1 (Thr37/46), 4EBP1 (Cell Signaling Technology, Danvers, MA, USA), β-actin (Sigma-Aldrich, St. Louis, MO, USA).

Microarray analysis

The microarray analysis was conducted at Shanghai Baygene Biotechnology Co. Ltd (Shanghai, China). Total RNA was extracted and subjected to gene expression analysis with Affymetrix Human PrimeView microarrays (Santa Clara, CA, USA) according to the manufacturer’s instructions. Molecular pathway analysis was performed using Gene Set Enrichment Analysis (GSEA, http://software.broadinstitute.org/gsea/index.jsp).

Immunofluorescence assay

Cells grown on coverslips were washed with phosphate-buffered saline (PBS) and fixed with 4% paraformaldehyde (Melonepharma, Dalian, China) for 15 min. Cells were then permeabilized with 0.2% Triton X-100 (Sigma-Aldrich) in PBS for 8 min, blocked with PBS containing 3% BSA for 30 min and incubated with antibodies against p53 overnight. After being washed with PBS, cells were incubated with Alexa Fluor 633-conjugated secondary antibodies (Invitrogen, Carlsbad, CA, USA) for 2 h, and then stained with DAPI (Vector Laboratories, Burlingame, CA, USA). Images of cells were acquired with Olympus BX51 fluorescence microscope (Olympus, Japan).

Nuclear/cytoplasmic fractionation

Nuclear/cytoplasmic fractionation was performed with Nuclear and Cytoplasmic Protein Extraction kit according to the manufacturer’s instructions (Beyotime Biotechnology, Shanghai, China). Equivalent amounts of protein of the nuclear or cytoplasmic lysates were subjected to Western blot.

Statistical analysis

Data presented were from at least two or three independent experiments. Statistical analysis was performed as indicated. Differences were considered statistically significant when p value was less than 0.05.

Supplementary Text

Abbreviations

PI3K, phosphoinositide 3-kinases;

HR, hormone receptor;

HER2, human epidermal growth factor receptor-2;

PTEN, gene of phosphate and tension homology deleted on chromosome ten;

mTOR, mammalian target of rapamycin;

CDK, cyclin-dependent kinase;

PIM, Proviral Insertion site in Murine leukemia virus kinase;

S6K1, ribosomal protein S6 kinase beta-1;

p-S6K1, phosphorylated S6K1;

mTORC1, mTOR complex 1;

GSEA, Gene set enrichment analysis;

GI_50_, the concentrations for 50% of maximal inhibition of cell proliferation;

XPO1, exportin 1;

4EBP1, eukaryotic translation initiation factor 4E binding protein 1;

p-4EBP1, phosphorylated 4EBP1;

CI, Combination Index.

Acknowledgements

This work was supported by "Personalized Medicines-Molecular Signature-based Drug Discovery and Development", Strategic Priority Research Program of the Chinese Academy of Sciences (XDA12020111); National Science and Technology Major Project “Key New Drug Creation and Manufacturing Program” (2018ZX09711002-011-014 & 2018ZX09711002-004-011) and National Natural Science Foundation of China (81773760). It was also partially supported by the Fudan-SIMM Joint Research Fund (FU-SIMM20172005).

Figure. S1.


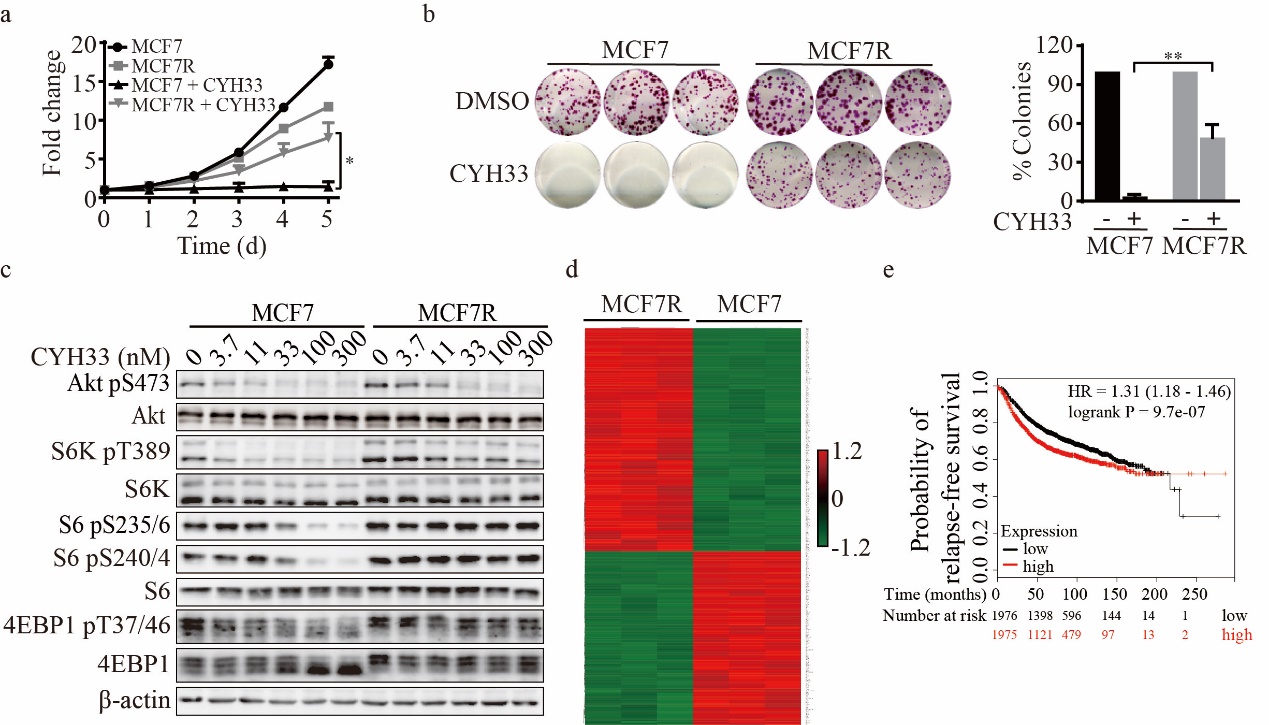


(a) Cells were treated with CYH33 (1 μM) or DMSO and cell proliferation was assessed by an SRB assay at indicated times. (b) Representative images of colony formation of indicated cells treated with CYH33 (0.3 μM) or DMSO and the colonies formed were quantitated. (c) Cells were treated with CYH33 for 24 h and cell lysates were subjected to Western blot with indicated antibodies. (d) The heatmap of genes differentially expressed with statistical significance (fold change cutoff of 1.5 folds) in MCF7R and parental cells. Biological replicates = 3. (e) The relationship between the expression level of XPO1 and the probability of relapse-free survival (RFS) in breast cancer patients was analyzed with Kaplan-Meier Plotter. Data shown in (a, b) are mean + SD. p value in (a, b) was calculated by Student’s t-test. **p < 0.01; *p < 0.05.

Figure. S2.


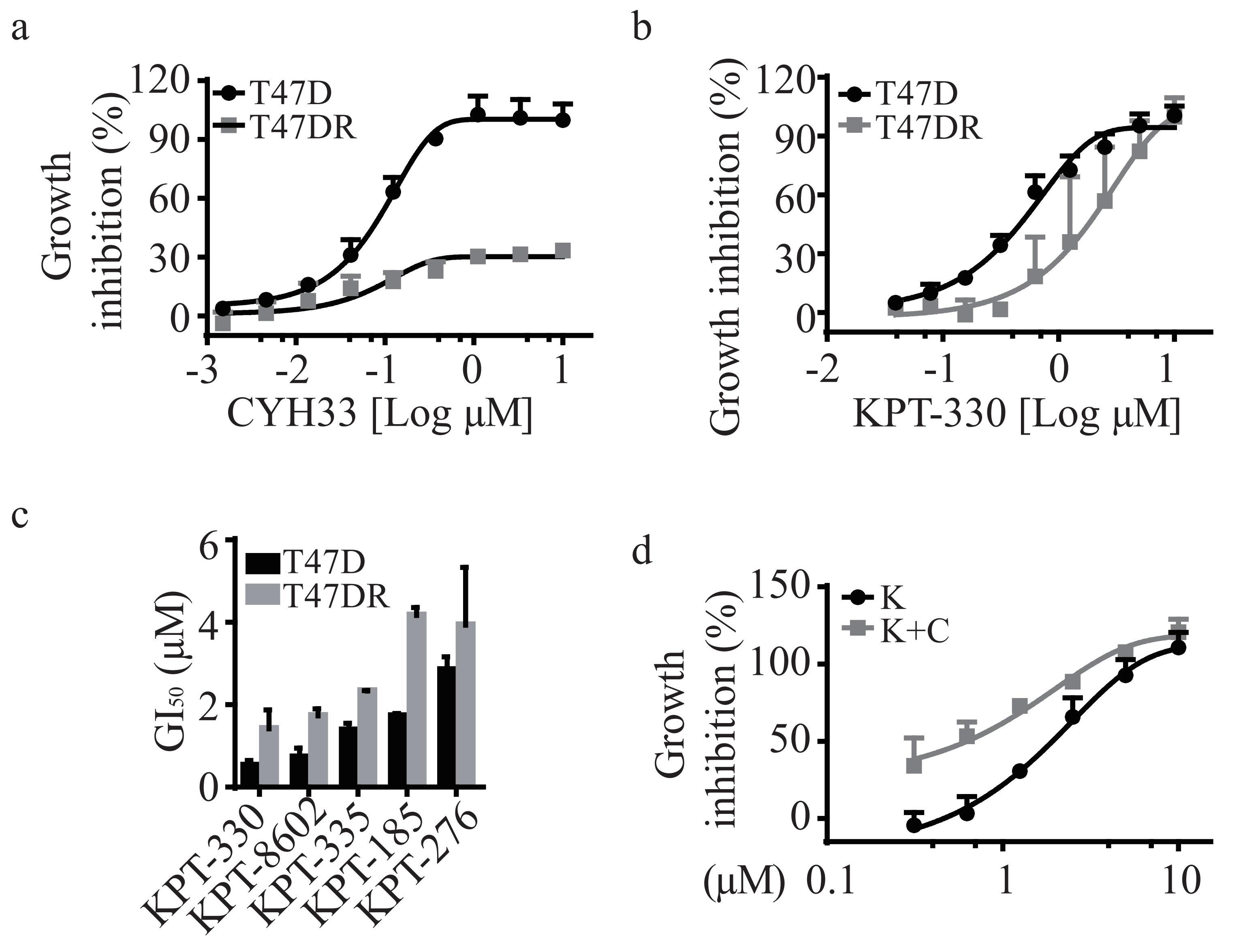


(a) The proliferation of indicated cells treated with CYH33 was evaluated with an SRB assay. (b) The effect of KPT-330 on the proliferation of indicated cells was evaluated with an SRB assay. (c) Cells were treated with indicated XPO1 inhibitors and GI_50_s were evaluated with an SRB assay. (d) Cells were treated with KPT-330 at various concentrations alone or concurrently with CYH33 (0.3 μM) for 72 h, and cell proliferation was measured with an SRB assay. Data presented were representative as mean + SD from at least three independent experiments. K: KPT-330; K+C: KPT-330 + CYH33.

Figure. S3.


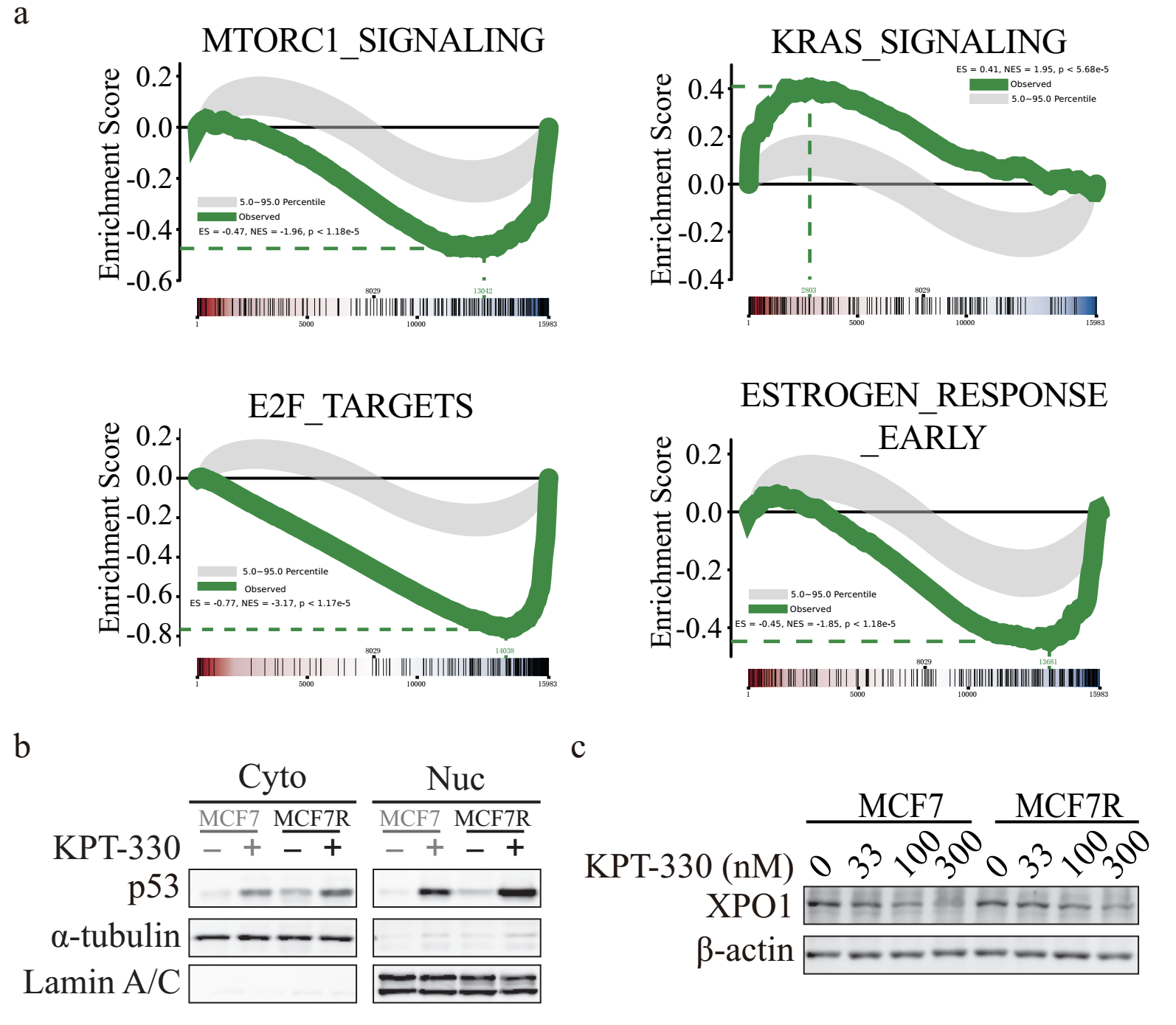


(a) Gene sets enriched in MCF7R cells with KPT-330 treatment compared to those in MCF7R cells were analyzed with GSEA. (b) Cells were treated with KPT-330 (1 μM) for 24 h and cells were collected for nuclear/cytoplasmic fractionation and Western blot with indicated antibodies. Cyto: cytoplasmic fractionation; Nuc: nuclear fractionation. Lamin A/C and α-tubulin were employed as loading control for nuclear or cytosolic protein respectively. (c) Cells were treated with KPT-330 (1 μM) for 24 h and cell lysates were processed for Western blot with indicated antibodies. Data presented were representative from at least three independent experiments.
